# Association of the D repeat polymorphism in the *ASPN *gene with developmental dysplasia of the hip: a case-control study in Han Chinese

**DOI:** 10.1186/ar3252

**Published:** 2011-02-17

**Authors:** Dongquan Shi, Jin Dai, Pengsheng Zhu, Jianghui Qin, Lunqing Zhu, Hongtao Zhu, Baocheng Zhao, Xusheng Qiu, Zhihong Xu, Dongyang Chen, Long Yi, Shiro Ikegawa, Qing Jiang

**Affiliations:** 1 The Center of Diagnosis and Treatment for Joint Disease, Drum Tower Hospital Affiliated to Medical School of Nanjing University, Zhongshan Road 321, Nanjing 210008, Jiangsu, PR China; 2 Laboratory for Bone and Joint Diseases, Model Animal Research Center, Nanjing University, Xuefu Road 12, Nanjing 210008, Jiangsu, PR China; 3 Center of Diagnosis and Treatment for Congenital Dysplasia of Hip, Kang'ai Hospital, Nanchang Road 32, Nanjing 210008, Jiangsu, PR China; 4 Department of Pathology, Medical School of Nanjing University, Hankou Road 22, Nanjing 210093, Jiangsu, PR China; 5 Laboratory for Bone and Joint Diseases, Center for Genomic Medicine, RIKEN, 4-6-1 Shirokanedai, Minato-ku, Tokyo 108-8639, Japan

## Abstract

**Introduction:**

Developmental dysplasia of the hip (DDH) is a common skeletal disease, which is characterized by abnormal seating of the femoral head in the acetabulum. Genetic factors play a considerable role in the etiology of DDH. Asporin (ASPN) is an ECM protein which can bind to TGF-β1 and sequentially inhibit TGF-β/Smad signaling. A functional aspartic acid (D) repeat polymorphism of *ASPN *was first described as an osteoarthritis-associated polymorphism. As TGF-β is well known as an important regulator in the development of skeletal components, *ASPN *may also be involved in the etiology of DDH. Our objective is to evaluate whether the D repeat polymorphism of *ASPN *is associated with DDH in Han Chinese.

**Methods:**

The D repeat polymorphism was genotyped in 370 DDH patients and 445 control subjects, and the allelic association of the D repeat was examined.

**Results:**

From D11 to D18, eight alleles were identified. D13 allele is the most common allele both in control and DDH groups, the frequencies are 67.3% and 58.1% respectively. In the DDH group, a significantly higher frequency of the D14 allele and significantly lower frequency of D13 was observed. The association of D14 and D13 was found in both females and males after stratification by gender. There was no significant difference in any other alleles we examined.

**Conclusions:**

Our results show an obvious association between the D repeat polymorphism of *ASPN *and DDH. It indicates that *ASPN *is an important regulator in the etiology of DDH.

## Introduction

Developmental dysplasia of the hip (DDH; MIM 142700) is a common skeletal disease, which is characterized by abnormal seating of the femoral head in the acetabulum [1]. The incidence of DDH varies from 1 per 1,000 to 18.4 per 1,000 in the Caucasian population, and in the Chinese the incidence of DDH is about 4 per 1,000 [1,2]. DDH could lead to early onset of hip osteoarthritis because of increased contact pressure between the acetabulum and femoral head [3-5]. Shallow acetabulum and lax capsule were considered to be the main causes of DDH [6,7]. Several family studies indicated that a considerable genetic component played an important role in the etiology of DDH [8-10]. A genome-wide screening from a large four-generation Japanese family of acetabular dysplasia had revealed a linkage between DDH and a specific region at chromosome 13 [11]. We had detected a definite association between a functional SNP in *GDF5 *and DDH by a case-control study in the Chinese population, and this association was also found in Caucasians [12,13].

Asporin (ASPN) is an ECM protein which belongs to the family of small leucine-rich repeat proteins [14]. Previous studies indicated that ASPN could bind to TGF-β1 and block its interaction with the TGF-β type II receptor, then sequentially inhibit the TGF-β/Smad signaling and TGF-β1 induced chondrogenesis [15,16]. TGF-β1 was a crucial regulator for the perichondrial cells and fibroblast cells in tendons. Binding to TGF-β1 may also inhibit perichondrium dependent skeletal development as well as development of tendons and ligaments [17,18]. ASPN can also bind to (bone morphogenetic protein 2) BMP2 and inhibit BMP/Smad signaling [19,20]. BMP2 is another growth factor of the TGF-β family which plays a general role in differentiation and proliferation of perichondrial cells and osteoblast [21,22].

Recently, an aspartic acid repeat polymorphism of *ASPN *was first described as an osteoarthritis-associated polymorphism. The D14 allele of *ASPN *was over-represented in osteoarthritis subjects, and D14 allele showed greater inhibition of TGF-β1 activity than the common allele, D13 [15]. This association was replicated in different populations and confirmed by meta-analysis although some studies denied this association [23-29]. This polymorphism was also identified to be associated with lumbar-disc degeneration and the outcome of rheumatoid arthritis [30,31].

As this polymorphism showed definite associations with various skeletal diseases [23-31], D14 allele and D13 allele of this polymorphism exhibited a remarkable difference in blocking TGF-β/Smad signaling [15]. We suspected that this polymorphism may also play a pivotal role in the etiology and pathogenesis of DDH. To evaluate the possible association, we conducted a case-control study on *ASPN *with DDH in the Chinese Han population and found a compelling association between *ASPN *and DDH.

## Materials and methods

### Subjects

A total of 756 subjects were studied. Of these, 370 patients (313 females and 57 males) were enrolled at the Center of Diagnosis and Treatment for DDH, Kang'ai Hospital, while 445 healthy control subjects (290 females and 155 males) were enrolled at the Physical Examination Center, Drum Tower Hospital, affiliated to the Medical School of Nanjing University. All subjects studied in the study were Chinese Han living in and around Nanjing. No subjects dropped out during the process of the study. The study was approved by the ethical committee of the participating institutions, and informed consent was obtained from all subjects. Patients were diagnosed by expert medical examination with radiographic evidence, and they all suffered from unilateral or bilateral DDH. Severity of DDH was defined from mild instability of the femoral head with slight capsular laxity, through moderate lateral displacement of the femoral head, without loss of contact of the head with the acetabulum, up to complete dislocation of the femoral head from the acetabulum [32]. Control subjects were identified by detailed inquiry of history and physical examination, and they never had any history or symptoms of DDH. Subjects with any systemic syndrome were excluded from this study. The ages of patients and controls (mean ± standard deviation (SD)) were 21.3 ± 12.2 (range, 2 to 51) months and 57.5 ± 11.9 (range, 40 to 97) years, respectively. The ratio of female to male was about 6:1 in these cases.

### Genotyping

Genomic DNA was extracted from peripheral blood using the Chelex-100 method or from buccal swabs using the DNA IQ System (Promega, Madison, WI, USA) according to the manufacturer's instructions [33]. DNA was genotyped for the *ASPN *microsatellite encoding the D repeat polymorphism after PCR amplification, the primers and thermal conditions were described before [23]. PCR products with 2 μL STR 2×Loading Solution (Promega) were loaded onto 6% denaturing polyacrylamide gel (BIO-RAD Sequi-Gen GT System 38 × 30 cm, CAT. No.165-3862, Hercules, CA, USA). Samples were run at 50°C for about two hours. After electrophoresis, the gels were stained with silver nitrate. Allele size determination was carried out by comparison to an allele ladder.

### Statistical analysis

Fisher's exact test was used to compare the *ASPN *genotype distribution in the case-control study. We assessed association and the Hardy-Weinberg equilibrium by the χ2 test. Odds ratio (OR), *P*-value and 95% confidence interval (CI) were calculated with respect to the minor allele compared with the major allele. Stratification analyses by gender of DDH were performed using SPSS 12.0 system software (IBM SPSS, Chicago, IL, USA).

## Results

Eight different alleles were identified, corresponding to 11 to 18 D repeats (Table 1). There were 21 genotypes; distributions of genotypes in the DDH and control groups were conformed to Hardy-Weinberg equilibrium (*P *= 0.723, *P *= 0.179, respectively). D13 was the most common allele in both patients and controls.

**Table 1:** Allelic frequency of the D-repeat polymorphism of *ASPN *in DDH in a Han Chinese population

Group	No. of subject	No. of allele (%)								
		
		D11	D12	D13	D14	D15	D16	D17	D18	Total
DDH										
All	370	1 (0.1)	168 (22.7)	430 (58.1)	70 (9.5)	23 (3.1)	43 (5.8)	5 (0.7)	0	740
Female	313	1 (0.2)	131 (20.9)	372 (59.4)	64 (10.2)	20 (3.2)	35 (5.6)	3 (0.5)	0	626
Male	57	0	37 (32.4)	58 (50.8)	6 (5.2)	3 (2.6)	8 (7.0)	2 (1.8)	0	114
CONTROL										
All	445	0	167 (18.8)	599 (67.3)	48 (5.4)	30 (3.4)	39 (4.4)	4 (0.4)	3 (0.3)	890
Female	290	0	110 (19.0)	391 (67.4)	32 (5.5)	16 (2.8)	29 (5.0)	1 (0.2)	1 (0.2)	580
Male	155	0	57 (18.3)	208 (67.1)	16 (5.1)	14 (4.5)	10 3.2)	3 (1.0)	2 (0.6)	310

*ASPN*, aspirin; D-repeat, aspartic acid repeat; DDH, developmental dysplasia of the hip.

In the DDH group, the D14 allele had a significantly higher frequency and the D13 allele had a significantly lower frequency. A significant difference in the allelic frequency was observed in comparison of D14 versus (vs.) other alleles combined (*P *= 0.0016), D13 vs. other alleles combined (*P *= 1.3*10^-4^) and D14 vs. D13 (*P *= 2.7*10^-4^) (Table 2). Considering eight alleles were tested (D11 to D18), then the Bonferroni corrected *P*-value should be 0.00625. The significance remained after applying the Bonferroni correction. No significant differences were observed in any other alleles for comparisons of one allele vs. all the remaining alleles combined.

**Table 2:** Association of the D-repeat of *ASPN *in patients with DDH in a Han Chinese population

Groups compared	D14 vs. Others			D13 vs. Others			D14 vs. D13		
			
	OR	*P-*value	95% CI	OR	*P-*value	95% CI	OR	*P-*value	95% CI
All patients (*n *= 370) vs. All controls (*n *= 445)	1.83	0.0016	1.25 to 2.68	0.67	1.3*10^-4^	0.55 to 0.82	2.03	2.7*10^-4^	1.38 to 2.99
Female patients (*n *= 313) vs. Female controls (*n *= 290)	1.95	0.0025	1.26 to 3.03	0.71	0.004	0.56 to 0.90	2.10	9.3*10^-4^	1.34 to 3.29
Male patients (*n *= 57) vs. Male controls (*n *= 155)	1.02	0.96	0.39 to 2.68	0.51	0.002	0.33 to 0.79	1.34	0.55	0.50 to 3.59

*ASPN*, asporin; CI, confidence interval; D-repeat, aspartic acid repeat; DDH, developmental dysplasia of the hip; OR, odds ratio.

We stratified subjects by gender and compared the allelic frequency. In female subjects, significant differences were observed in a comparison of D14 vs. other alleles combined (*P *= 0.0025), D13 vs. other alleles combined (*P *= 0.004) and D14 vs. D13 (*P *= 9.3*10^-4^) (Table 2). A significant difference was detected in comparison of D13 vs. the other alleles combined in males (*P *= 0.002) (Table 2). The significance remained after applying the Bonferroni correction. No significance was found in other alleles for comparisons of one allele vs. all remaining alleles combined after stratification of gender.

## Discussion

We have demonstrated *ASPN *as a susceptibility gene of DDH with a case-control association study in Chinese Han population. D14 was identified as the risk allele; otherwise the common allele, D13, seemed to be a protective allele. Association was detected in both female and male subjects after stratification by gender.

A detailed analysis of ASPN expression in embryonic and adult mouse limbs showed that ASPN was expressed in perichondrium, periosteum, fascia, and tendon, but not in the articular cartilage and growth plate cartilage [34]. We considered that this polymorphism was not involved in the process of chondrocyte differentiation, although ASPN was demonstrated to inhibit chondrogenesis and chondrogenic differentiation via TGF-β/Smad signaling in both mouse and human cell lines [16].

TGF-β and BMP2 were crucial for the differentiation and proliferation of perichondrial cell and fibroblast cells [17,18,21]. Inhibition of TGF-β/Smad and BMP2/Smad signaling may reduce the differentiation and proliferation of perichondrial cells, and then delay the development of skeletal components; and it may also deduce the proliferation of fibroblast cells in tendon and fascia, and then loosen the tendon and fascia around a joint, which will make the joint easier to be dislocated.

D14 allele had a significant higher inhibitory effect on TGF-β signaling [15], it may contribute to the susceptibility of DDH via one or both of these two mechanisms, defected soft tissues around hip joint and delayed skeletal development of the hip joint. On the other hand, the D13 allele had a significant weaker inhibition on TGF-β signaling, so it exhibited a protective role in the pathogenesis of DDH.

## Conclusions

Our study suggested an association of *ASPN *with DDH susceptibility in a Chinese Han population, and ASPN is an important regulator in pathology of DDH. It may influence the susceptibility of DDH via TGF-β signaling.

## Abbreviations

ASPN: asporin; BMP2: bone morphogenetic protein 2; D: aspartic acid; DDH: developmental dysplasia of the hip; TGF-β: transforming growth factor-β.

## Competing interests

The authors declare that they have no competing interests.

## Authors' contributions

All authors contributed to the final manuscript. In addition, DS and JD genotyped the samples and participated in the design and analysis of the study. PZ, JQ, LZ, HZ, BZ, XQ, ZX and DC evaluated the patients and genotyped these samples. LY and SI coordinated the study. QJ supervised the whole study.
